# Phosphorus Recovery from Sewage Sludge Using Acidithiobacilli

**DOI:** 10.3390/ijerph18137135

**Published:** 2021-07-03

**Authors:** Surendra K. Pradhan, Helvi Heinonen-Tanski, Anna-Maria Veijalainen, Sirpa Peräniemi, Eila Torvinen

**Affiliations:** 1 Department of Environmental and Biological Sciences, University of Eastern Finland, FI-70211 Kuopio, Finland; helvi.heinonentanski@uef.fi (H.H.-T.); anna-maria.veijalainen@uef.fi (A.-M.V.); eila.torvinen@uef.fi (E.T.); 2 Department of Pharmacy, University of Eastern Finland, FI-70211 Kuopio, Finland; sirpa.peraniemi@uef.fi

**Keywords:** *Acidithiobacillus thiooxidans*, *Acidithiobacillus ferrooxidans*, bioleaching, phosphorus solubilization, recovery, sewage sludge

## Abstract

Sewage sludge contains a significant amount of phosphorus (P), which could be recycled to address the global demand for this non-renewable, important plant nutrient. The P in sludge can be solubilized and recovered so that it can be recycled when needed. This study investigated the P solubilization from sewage sludge using *Acidithiobacillus thiooxidans* and *Acidithiobacillus ferrooxidans*. The experiment was conducted by mixing 10 mL of sewage sludge with 90 mL of different water/liquid medium/inoculum and incubated at 30 °C. The experiment was conducted in three semi-continuous phases by replacing 10% of the mixed incubated medium with fresh sewage sludge. In addition, 10 g/L elemental sulfur (S) was supplemented into the medium in the third phase. The pH of the *A. thiooxidans* and *A. ferrooxidans* treated sludge solutions was between 2.2 and 6.3 until day 42. In phase 3, after supplementing with S, the pH of *A. thiooxidans* treated sludge was reduced to 0.9, which solubilized and extracted 92% of P. We found that acidithiobacilli supplemented with S can be used to treat sludge, i.e., achieve hygienization, removal of heavy metals, and solubilization and recovery of P.

## 1. Introduction

Phosphorus (P) is an important plant nutrient and an increasingly valuable natural resource due to its limited supply. On the other hand, P leaching to surface water is often a serious environmental problem as noticed, for instance, in the Baltic Sea [1]. Sewage sludge contains a significant amount of P. A large amount of sewage sludge is generated worldwide every day and the amounts of sludge are expected to increase as a consequence of population growth. The disposal of such a large quantity of sewage sludge is a serious environmental concern. The spreading of sludge on agricultural land would be a good option since it recycles nutrients and increases soil organic matter. There are, however, several constraints to the reuse of sewage sludge in agriculture: (1) sludge contains a large number of pathogens, (2) it may contain high concentrations of heavy metals, and (3) it is bulky and expensive to transport. Despite these constraints, a significant amount of sludge is recycled in agriculture, which in turn, means that this is one of the main sources of P contamination in water bodies [1]. Therefore, the sewage sludge has to be treated properly before reuse.

P can be recovered from sewage sludge as phosphoric acid by using sulfuric acid [2], but drying the sludge and using a large amount of commercial acid is expensive. The bioleaching of rock phosphate with acid-producing microorganisms has been widely studied [3,4,5]. The advantages of the biochemical process are its low costs [6], high efficiency, and environmental friendliness. Therefore, it would be highly advantageous to develop a biochemical process to recover P from sludge.

The bacteria *Acidithiobacillus ferrooxidans* and *Acidithiobacillus thiooxidans* are autotrophic, mesophilic, and acidophilic iron- and sulfur-oxidizing bacteria [7]. They can produce sulfuric acid from reduced sulfur compounds (Equation (1) so that the pH can be even lower than 1 [3,8,9,10]. The produced sulfuric acid solubilizes the phosphorus (Equations (2) and (3) [4]. It has been reported that *A. thiooxidans* was able to recover 94% of P from rock phosphate [5]. In another investigation, *A. ferrooxidans* was used to solubilize P from dried sewage sludge [11].

2S^0^ + 3O_2_ + 2H_2_O→2H_2_SO_4_ (1)

3H_2_SO_4_ + Ca_3_(PO_4_)_2_→2H_3_PO_4_ + 3CaSO_4_ (2)

3H_2_SO_4_ + 2FePO_4_→2H_3_PO_4_ + Fe_2_(SO_4_)_3_ (3)

Sewage sludge was selected for this study since (1) it contains large amounts of P, (2) the production of sewage sludge is increasing, and (3) it can contain a high level of iron (Fe) and sulfate (SO_4_) since iron sulfate is used for coagulation in wastewater treatment plants.

The main aim of this study was to test the ability of both the *A.* *thiooxidans* and *A. ferrooxidans* to recover P from sewage sludge. The individual objectives were (1) to compare the abilities of the two acidophilic bacteria to solubilize the P from sewage sludge and (2) to identify the needs of these bacteria for optimal growth in a sludge environment.

## 2. Materials and Methods

### 2.1. Sewage Sludge

Partially dewatered sludge (about 5% total solids content) was collected from the Lehtoniemi wastewater treatment plant in Kuopio, Finland, and stored at 4 °C. The microbial and chemical characteristics of the sludge were analyzed before the experiment (Table 1).

### 2.2. Chemical Analysis

The physicochemical analysis of sludge was performed before and after the experiment. The pH was measured using an electrode (pH 3210, WTW, Weilheim, Germany). Total solid (TS) was analyzed by drying the sludge at 105 °C (Memmert UM 400 Drying Oven) for 24 h and organic matter (OM) was analyzed as volatile solids by dry combustion of the dried sludge at 550 °C for 2 h in an incinerator (Carbolite ELF 11/14). The total concentration of heavy metals, total-S (sulfur) and total-P were determined using total reflection X-ray fluorescence (TXRF) (S2 Picofox TXRF Spectrometer, Bruker, Hanau, Germany) after acid digestion. The total concentrations of heavy metals were measured from the sludge (before the experiment) and the supernatants of the sludge solution at the end of each of the three phases of the experiment. The chemical characteristics of the dewatered sludge samples before treatment are presented in Table 1. Total-N, PO_4_-P, soluble iron, sulfate, and total-P were analyzed with a Hach spectrophotometer according to the standard method described in the company’s catalog. Total-P was also determined by the spectrophotometer using the standard method [12].

### 2.3. Fecal Indicator Bacteria

Fecal coliforms, total coliforms, clostridia, and enterococci were analyzed before and after each phase of the treatment. Fecal coliforms were determined using the standard plate count method on MFC agar and incubated (Termaks, Bergen, Norway) at 44 °C for 24 h [13]. Enterococci were cultured in bile esculin azide agar and incubated at 37 °C for 48 h [14]. Clostridia were determined on sulfite-iron agar after the sample was treated at 80 °C for 10 min and the agar plates were incubated anaerobically at 37 °C for 48 h [15]. The typical bacterial colony forming units (CFU) were counted from plates and calculated as log_10_ CFU/mL (Table 1). A similar method was used to analyze these bacteria from sludge after treatment and the results are presented as log_10_ CFU/mL.

### 2.4. Microorganisms and Growth Media

The liquid cultures of *Acidithiobacillus ferrooxidans* (DSM 29444) and *Acidithiobacillus thiooxidans* (DSM 14887) were purchased from DSMZ, Germany. The cells were further inoculated in liquid media according to the instructions provided by the company.

*A. ferrooxidans* cultures were grown in DSMZ 882 liquid medium. The medium was prepared by mixing solution A (950 mL), B (49 mL), and C (1 mL) (Solution A: (NH_4_)_2_SO_4_ 132 mg/L, KH_2_PO_4_ 27 mg/L, CaCl_2_ × 2H_2_O 147 mg/L, MgCl_2_ × 6H_2_O 53 mg/L. Solution B: FeSO_4_ × 7H_2_O 20g/L. Solution C: MnCl_2_ × 2H_2_O 76 mg/L, ZnCl_2_ 68 mg/L, CoCl_2_ × 6H_2_O 64 mg/L, H_3_BO_3_ 31 mg/L, Na_2_MoO_4_ 10 mg/L, CuCl_2_ × 2H_2_O 67 mg/L) according to the instructions provided by DSMZ [16]. The agar plates for this bacterium were prepared by mixing the solutions A (600 mL), B (150 mL), and C (250 mL) (Solution A: (NH_4_)_2_SO_4_ 3 g/L, KCl 100 mg/L, KH_2_PO_4_ 50 mg/L, MgSO_4_ × 7 H_2_O 500 mg/L, Ca(NO_3_)_2_ × 4H_2_O 1.5 mg/L. Solution B: FeSO_4_ × 7 H_2_O 22 g/L. Solution C: Agarose 15 g/L) as described by Visca [17].

Cultures of *A. thiooxidans* were grown in DSMZ 71 liquid medium; this medium was prepared by mixing solutions A (900 mL) and B (100 mL) (Solution A: (NH_4_)_2_SO_4_ 3 g/L, KH_2_PO_4_ 3 g/L, CaCl_2_ × 2 H_2_O 0.25 g/L, MgSO_4_ × 7H_2_O 0.5 g/L, Solution B: Na_2_S_2_O_3_ × 5H_2_O 3.2 g/L) according to the instructions given by DSMZ [18]. The agar plates for *A. thiooxidans* were prepared by mixing solutions A (850 mL), B (148 mL), and BCG (2 mL) (Solution A: (NH_4_)_2_SO_4_ 0.4 g/L, MgSO_4_ × 7 H_2_O 0.5 g/L, CaCl_2_ 0.25 g/L, KH_2_PO_4_ 4 g/L, FeSO_4_ 0.01 g/L, Agar-2 13 g/L. Solution B: Na_2_S_2_O_3_ × 5H_2_O 5 g/L, BCG (bromocresol green) (0.4% solution) 2 mL/L) as described [19]. All these prepared media were stored at 4 °C and used within two months.

### 2.5. Cultivation and Enumeration of Acidithiobacilli

Each *Acidithiobacillus* spp. was cultivated in 1 L of its respective DSMZ medium at 30 °C for 15 days following the instructions provided by DSMZ. The bacterial numbers in the inoculum were determined by direct count, spread plate, and the MPN (most probable number) method.

In the direct count method, a few drops of Trypan solution (Trypan Blue stain 0.4%, Invitrogen, Molecular Probes, Lawrence, Massachusetts, USA) were added to the diluted sample to differentiate between viable and dead cells. The cells were counted under a light microscope using the Fuchs Rosenthal counting chamber (Germany). The observed cells are presented as log_10_ cells/mL (Table 2). In the agar plate culture method, 0.1 mL of sample from different dilutions was cultured on agar plates using the spread plate method and incubated at 30 °C for 10–15 days. These colonies were counted with the density calculated as log_10_ CFU/mL (Table 2). In the MPN method, 0.1 mL of the sample was added to 5 tubes with cultivation media and incubated at 30 °C for 10–15 days. The positive and negative tubes were observed, and the results were interpreted using the MPN table. The MPN results are presented in Table 2.

The acidophilic microorganisms were also analyzed after each experimental phase using the spread plate culture method as described for the inoculum.

### 2.6. P-Solubilization Experimental Setup

A total of 90 mL of the sterilized broths (growing media)/inocula were poured into 250 mL glass bottles and 10 mL of sewage sludge was added. The solutions were mixed well and incubated at 30 °C in a shaker incubator at 150 rpm (Certomat shaker and incubator). The bottles were closed loosely to guarantee good aeration. The details of the experimental setup and symbols are given in Table 3. The pH was measured two to three times each week during the incubation.

The experiment was designed as a semi-continuous process and conducted in three phases. The incubation of phase 1 lasted for 21 days. On day 21, 10 mL of the sample was removed from the incubated bottle and 10 mL of the new sludge was added. Phase 2 was conducted from day 21 to day 42. As expected, the pH did not decline until the end of phase 2 (day 42), therefore elemental sulfur was supplemented in phase 3. At the start of phase 3 or on day 42, 10 mL of the incubated sample was replaced with 10 mL of new sludge, and 10 g/L of elemental sulfur (S) (Fluka 80% pure) was added. The incubation then continued until day 63 when the experiment was terminated.

After the experiment, indicator microorganisms and acidophilic bacteria were analyzed from the incubated sludge solution and the result is presented in Table 4. The remaining solutions were filtered through a 0.45 µm filter paper and separated into solid and liquid fractions. The pH, total-P, PO_4_-P, total-S, SO_4_, total iron, Fe^2+^, and trace elements were determined from the liquid fraction. The soluble elements in the solutions were calculated from the original sludge and presented as soluble percentages (Table 5).

### 2.7. Statistical Analyses

All the raw data were gathered in an Excel file and transformed into the SPSS 25 (IBM, Armonk, NY, USA). These data were tested for normality before statistical analysis. The normally distributed data were analyzed using One-Way ANOVA combined with Tukey’s post hoc tests. The pH and percentage of solubilized elements were analyzed using Pearson and Spearman correlations.

## 3. Results and Discussion

### 3.1. Role of Acidithiobacilli

**Figure 1:**
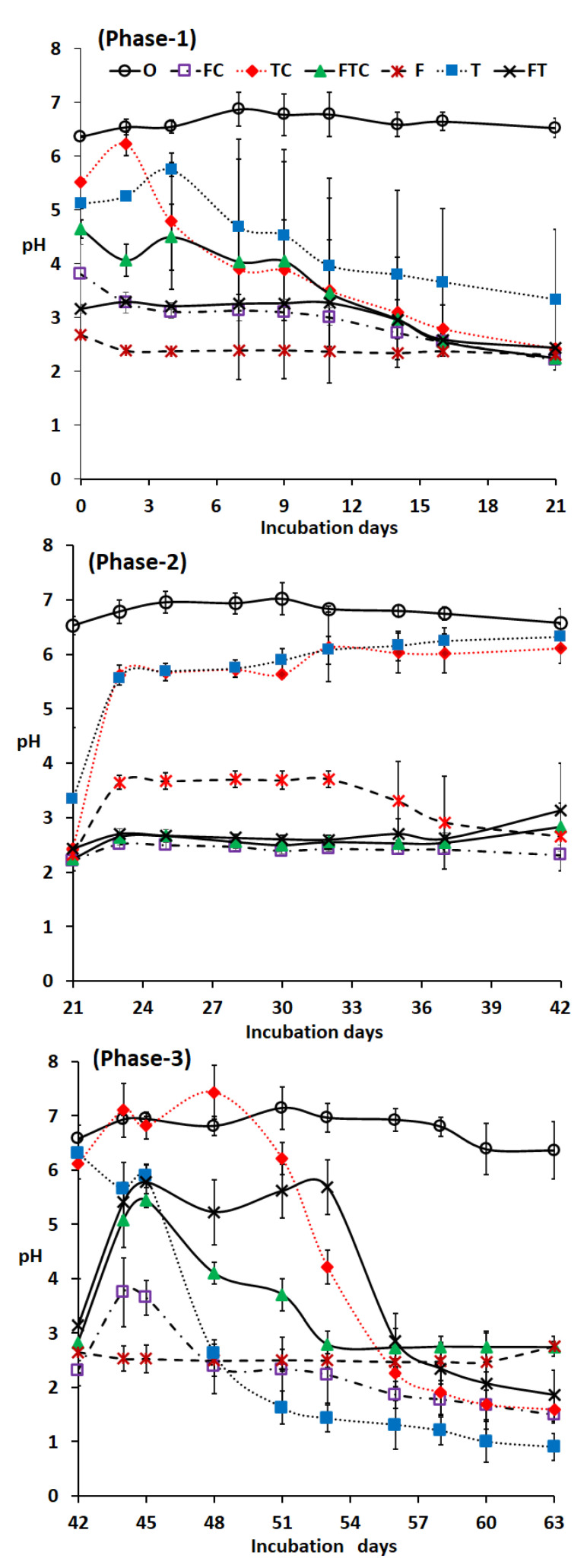
The pH of the incubated solution with different treatments in phases 1, 2, and 3. In phase 3 or 42 days of incubation, 10 mL of sample was replaced with 10 mL of new sludge +1 g of S to 100 mL. The symbols describing the treatments are presented in Table 3.

This study showed that *A. ferrooxidans* and *A. thiooxidans* pure and mixed cultures survived very well in the sewage sludge for 63 days at 30 °C (Table 4). The *A. thiooxidans* population was higher at the end of phase 1 as compared to the situation at the end of phase 2 in the TC and T treatments. This might be due to the residual soluble sulfate in the liquid media and inoculum. Another reason might be that the low pH had solubilized the sulfate present in sludge. In phase 2, the exchange of 10 mL of sludge increased the pH of the solution (Figure 1) and the higher pH might have reduced the solubility of sulfate, which in turn might have inhibited the growth of *A. thiooxidans*. It is recognized that the growth of acidophilic bacteria may be inhibited by the increasing total solid content [20]. Our result showed that total solid (TS) up to 1.1% (*w*/*v*) did not affect the growth of acidophilic bacteria. More work will be needed to identify the range of TS in the sludge inoculum that can achieve better acidophilic bacterial growth.

After the addition of S in phase 3, the growth of *A. thiooxidans* was increased in treatments T and TC (Table 4), whereas their pH was reduced. The decrease of media pH is an indirect indication of the growth of the acidophilic bacteria and their production of sulfuric acid [21,22]. Our result was similar to that presented by Pangayao et al. [22], who used the same S treatment.

It was found that unidentified S- and Fe-oxidizing microbes were already present in the used sludge and that these microbes grew in the control liquid media (i.e., TC, FC and FTC). This might be attributable to the presence of nutrients and optimum pH in liquid media but not in the water control (0-Control in Table 3). These indigenous sulfur- and/or iron-oxidizing microbes that had originated from the sludge grew well in our selective agar plates, which made it easier to detect their presence. Unfortunately, molecular identifications were not performed for these indigenous acidithiobacilli, so that we do not know how closely related they are to the *A. ferrooxidans* and *A. thiooxidans* used in our study.

### 3.2. The pH in the Experimental Sludge Solution

In this process, the pH was found to be the major factor determining the solubilizing of the P and heavy metals and hygienization of the sludge. The initial pH of the sludge was 6.5 ± 0.4 (Table 1) and the pH of the control (O) remained similar during the entire experiment, even after the addition of S (Figure 1). The pH values of the *A. thiooxidans* treated sludge and liquid media control (TC, T and FT) were increased on days 1–4 in phase 1 of the experiment, possibly because the calcium in sludge may have released some alkalinity [23]. At the beginning of the incubation, treatments T and TC had pH > 5. The pH values gradually declined until the end of phase 1 but they increased in phase 2 (Figure 1). This might be due to the residual S from liquid media/inoculum in phase 1 but the lack of a sufficient level of S compounds in phase 2 meant that the production of sulfuric acid was low. Another reason might be that the sulfate in sludge could no longer be solubilized in phase 2 since the pH of the *A. thiooxidans* treated sludge had increased to >pH 6 after the exchange of sludge at the beginning of phase 2.

*A. ferrooxidans* liquid medium and inoculum treated sludge, i.e., FC and F had significantly lower pH values (*p* < 0.05) compared to *A. thiooxidans* liquid medium and inoculum treated sludge, i.e., TC and T in phases 1 and 2. This might be because of the lower pH of the *A. ferrooxidans* liquid medium and inoculum (i.e., about pH 2) than that of the *A. thiooxidans* inoculum (i.e., about pH 4). The other reason for the low pH might be that the *A. ferrooxidans* was likely to simultaneously oxidize both iron and sulfur compounds, thus increasing the acidity [24]. However, *A. ferrooxidans* metabolizes slowly so sulfur oxidization would also be slow, and this bacterium produces a very small amount of sulfuric acid [24]. After adding S in phase 3, *A. thiooxidans* reduced the pH more than *A. ferrooxidans* or the *A. ferrooxidans* and *A. thiooxidans* mixture. A similar result has been reported in a past study [3] when *A. thiooxidans* produced more acids (39.81 mM H_2_SO_4_) than *A. ferrooxidans* alone (28.83 mM H_2_SO_4_) or *A. ferrooxidans* and *A. thiooxidans* together, especially in the metabolism of elemental S [25].

After adding elemental S in phase 3, the pH of the sludge solution treated with *A thiooxidans* treatments (TC+S, FTC+S, T+S) decreased. The treatment T+S eventually reached the lowest pH of all the treatments. These low pH values may have been caused by the added S, which is oxidized to sulfuric acid by *A. thiooxidans* [26]. *A. thiooxidans* produced 0.125 M of H_2_SO_4_ in treatment T+S (calculated as H^+^ = 10^−pH^). A similar reduction of pH was evident in the liquid medium control media TC+S and FTC+S because these controls contained indigenous sulfur-oxidizing microbes (Table 4). However*,* these indigenous acidophilic microbes were not able to reduce the pH efficiently and thus the solubilization of P remained modest. After 63 days of incubation, the pH with S was significantly lower than the situation without S (*p* = 0.03, F = 5.77) (data not shown). However, it would be interesting to clarify if P-solubilization would be increased by S-additions already in phases 1 or 2 or at both of these time points. Although there is some debate about the optimum temperature for *A. thiooxidans*, 28–30 °C has been the most commonly used temperature [27], as applied in this study.

### 3.3. Solubilization of Major Nutrients and Trace Elements

**Figure 2:**
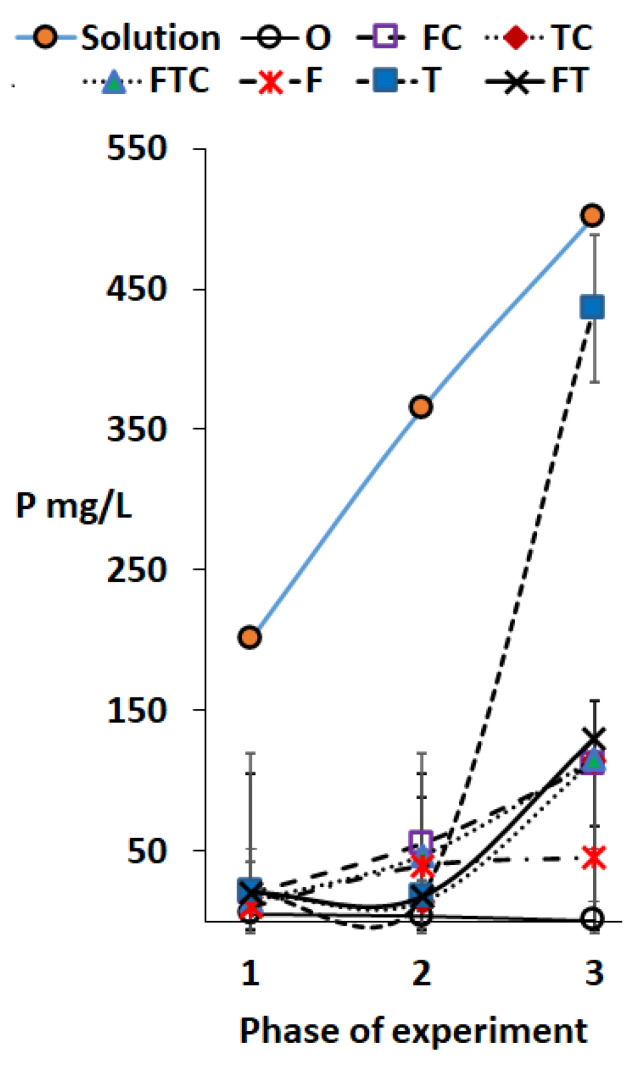
The total P in sludge at the beginning of each phase and the soluble PO_4_-P in supernatants (after filtration) at the end of the different phases of the study. The solution symbol in this figure resembles the total P in the incubated solution at the beginning of each phase.

The highest degree of P solubilization (92%) was observed at the pH of 0.9 in *A. thiooxidans* treated sludge, i.e., T+S in phase 3 (Table 5, Figure 2). The P solubilization was negatively correlated (*p* < 0.05, r = −0.802) with the pH of the sludge solutions and a significant amount of P solubilization occurred only at pH < 2. Generally, some P solubilization can start at pH as high as 5 but the extent of solubilization is modest until the pH reaches 2.5, especially if pH is adjusted by organic acids [28]. According to these authors, H_2_SO_4_ solubilizes less P than HNO_3_ and HCl if the same moles of acids are used. The P content in our reactor was 200 mg/L in phase 1, 366 mg/L in phase 2, and 501 mg/L in phase 3. Less than 20% of the P was solubilized when the pH of the sludge solution was above 2. A similar result has been reported earlier [26], but only 18% of P was solubilized with organic acids at pH 1.7.

Most of the P present in sludge may be present as FePO_4_ [29] or as different calcium (Ca) and hydrogen phosphates [30], which have a low solubility in weak acids. The clear reduction of pH in phase 3 was found after the addition of elemental S since *A. thiobacillus* (close to *A. thiooxidans*) oxidized elemental S to H_2_SO_4_ [10] and the sulfuric acid was able to solubilize the P in the sludge. A similar result was reported by Lee et al. [26], who achieved 48% P solubilization in 17 days of incubation at pH 0.9 using *A. thiooxidans* supplemented with S as applied here, but we achieved a higher P solubilization than these investigators. The higher P-solubilization in our study may have been due to the longer incubation time (i.e., 42 days or phases 1 and 2) prior to the addition of the S supplement, meaning that the acidophilic bacteria might have acclimatized better to the medium used here.

The sludge used in this study contained a high amount of Fe^2+^ and S (Table 1). The acidophilic bacteria are able to use these elements to make H_2_SO_4_ (Equation (1)) in accord with the earlier results [9]. A high amount of sulfur or reduced sulfur compound in the solution was converted into H_2_SO_4_ but there was still some residual sulfur in the solution (data not shown) as *A. thiooxidans* can oxidize only a limited amount of S [26,31]. It should be possible to reduce the amount of residual S by further optimization of the method. The extracted iron percentage was higher in treatments FC, FTC, F, and FT. This may be attributable to the use of a low pH inoculum and liquid medium (DSMZ 882) at the beginning, which solubilizes the iron. Another reason might be that iron is solubilized even at low acidity. For example, it has been reported that 36% of iron is solubilized at pH 6 with 100% being solubilized at pH 2 [32].

Most heavy metals tested were extracted in the liquid when the pH of the sludge decreased to about 2.5. A significant amount of rubidium (Rb) was extracted even at pH 7. The low pH values in phase 3 extracted most trace elements, i.e., chromium (Cr), manganese (Mn), cobalt (Co), nickel (Ni), copper (Cu), zinc (Zn), and strontium (Sr). The trace elements titanium (Ti) and bromine (Br) were solubilized to a lesser extent at very low pH values. The low pH increased the solubility of most of the elements. The pH in the solution was significantly negatively correlated with the solubilization of Ca, Ti, Cr, Mn, Co, Ni, Cu, Zn, and Sr (in each case *p* < 0.05 and correlation coefficients from −0.51 to −0.87). Compared to our result, the lower degree of solubilization of Zn (56.9%), Mn (70.9%), Cr (85.0%), Fe (74.4%), and Cu (74.5%) have been reported previously [22], which might be because the incubation time was only 15 days in that experiment. We observed that Zn, Cr, Mn, Fe, and Cu could be solubilized even at pH 3–4, as also found previously [33], but the solubilization of Cr started at pH 6. This finding can help in the choice of different treatments for different outputs. For example, if the requirement is to eliminate certain trace elements, *A. ferrooxidans* treatment (F) or *A. ferrooxidans* liquid medium control (FC) can be used since they decreased the sludge pH to about 2.5 within 21–42 days and solubilized a substantial amount of the trace elements (Table 6).

### 3.4. Hygienization of Sludge

The numbers of fecal coliforms and total coliforms decreased to less than detection limits (LDL) within 21 days of incubation in acidithiobacilli treated sludge (Table 1 and Table 4). This is expected since such a low pH does not favor the growth of most coliform bacteria [34]. The numbers of enterococci and clostridia gradually declined up to 42 days or at the end of phase 2, and they had been reduced to LDL by the end of phase 3 (Table 1 and Table 4). Enterococci and clostridia have a higher survival rate in different environments, and thus they survived in sludge for a long time even at pH 2.5, but these bacteria could not survive when the pH was lower than 2. It has been reported that the survival of some pathogens can be reduced at pH values lower than 3.5 [35], but the suppression of enterococci required a pH under 2.5 [36]. Clostridia, meanwhile, could not survive at a pH below 2 [37], in contrast with the results of our study (Table 4).

## 4. Conclusions

In conclusion, both acidophilic bacterial types could survive in sludge media at 30 °C for 63 days. *A. thiooxidans* was able to solubilize 92% of P of sewage sludge if the medium was supplemented with elemental S (1 g/100 mL). Oxidation of the supplemented S resulted in a highly acidic, i.e., pH 0.9, sludge solution. Large amounts of heavy metals were removed from the sludge. All acidophilic bacterial treatments had hygienized the sludge after 63 days of incubation. Although the method took 63 days to recover 92% of P, hygienize the sludge, and eliminate the trace elements, these activities mainly occurred in phase 3, i.e., 21 days of incubation after S addition. The extracted phosphoric acid (P solubilized acidic supernatant) can be used for different purposes including the production of ammonium phosphate. We conclude that the recovery of P from sludge will not only help to prevent the depletion of P natural resources but also improve food security.

## Figures and Tables

**Table 1:** Different parameters of the sludge sample before the experiment (without dilution). FW = fresh weight.

Chemical Parameters	Before the Experiment
pH	6.5 ± 0.4
Total solids (%)	5.59 ± 0.02
Organic matter (%)	5.16 ± 0.07
Chemical oxygen demand (g/L)	240
Total-P (g/L) FW	2
PO_4_-P (mg/L) FW	130
Total-N (g/L) FW	15.5
Total-sulfur (S) (mg/L)	840
K (mg/L)	14
Ca (mg/L)	79.2
Total-iron (Fe) (g/L)	9.1
Fe^2+^ (g/L)	1.5
**Indicator bacteria**	**Log_10_ CFU/mL**
Fecal coliforms	5.79 ± 0.57
Total coliforms	6.29 ± 0.25
Enterococci	4.19 ± 0.28
Clostridia	4.65 ± 0.32

**Table 2:** Numbers of acidithiobacilli in inocula before the experiment (mean ± st. dev). (N = 3).

Method	*A. ferrooxidans*	*A. thiooxidans*
Microscopic cell count (log_10_ cells/mL)	6.76 ± 0.15	6.87 ± 0.47
Agar plate (log_10_ CFU/mL)	7.07 ± 0.11	7.92 ± 1.06
MPN (log_10_ MPN/mL)	7.0	7.7

**Table 3:** Treatments and symbols. Symbols + S means that the sulfur was added to 100 mL solution only in phase 3. Sulfur (S).

Symbol	Treatment	Description
Before (S) supplement phases 1 and 2		
0	0 Control	Sewage sludge + sterile water
FC	F Control	Sewage sludge + DSMZ 882 liquid medium aimed for *A. ferrooxidans*
TC	T Control	Sewage sludge + DSMZ 71 liquid medium aimed *for A. thiooxidans*
FTC	FT Control	Sewage sludge + liquid medium (50% of DSMZ 882 and 50% of sterile DSMZ 71)
F	F Treatment	Sewage sludge + *A. ferrooxidans* inoculum
T	T Treatment	Sewage sludge + *A. thiooxidans* inoculum
FT	FT Treatment	Sewage sludge + bacterial inoculum (50% of *A. ferrooxidans* + 50% of *A. thiooxidans*)
After S supplement phase 3		
0 + S		0 control incubated mixture solution + S
FC + S		F Control incubated mixture solution + S
TC + S		T Control incubated mixture solution + S
FTC + S		FT Control incubated mixture solution + S
F + S		F Treatment incubated mixture solution + S
T + S		T Treatment incubated mixture solution + S
FT + S		FT Treatment incubated mixture solution + S

**Table 4:** Numbers of acidithiobacilli (originating from sludge and DSMZ collection) and fecal indicator bacteria at the end of each phase of the experiment (log_10_ CFU/mL). (N = 3). LDL (less than detection limit) is 100 CFU/mL. The numbers 1, 2 and 3 refer to the three phases of the experiment. Sulfur- and iron-oxidizing (S- and Fe-oxidizing). O is control (sterile water and sludge). FC is medium DSMZ 882 and TC is medium DSMZ 71 and FTC is a mixture of both DSMZ media. Used acidophilic bacteria F (*A. ferrooxidans*), T (*A. thiooxidans*), and FT (*A. ferrooxidans* and *A. thiooxidans*). Different letters in the same row are significantly different results (*p* < 0.05).

Microbes	Phase	O	FC	TC	FTC	Used Acidophilic Bacteria in F, T and FT Treatments	F	T	FT
S- and Fe-oxidizing microbes	1	5.0 ± 0.3 a	2.0 ± 0.2 b	5.1 ± 0.1 a	5.2 ± 0.1 a	*A. ferrooxidans*	4.2 ± 0.2 a	7.7 ± 0.1 c	4.0 ± 0.2 a
S- and Fe-oxidizing microbes	2	2 ± 0.2 a	5 ± 0.5 b	5.9 ± 0.5 b	6 ± 0 b	*A. ferrooxidans*	6 ± 0 b	7 ± 0.7 b	5 ± 0.9 b
S- and Fe-oxidizing microbes	3	0.7 ± 0.4 a	10.0 ± 1.0 b	6.5 ± 0.3 c	8.0 ± 0.0 bc	*A. ferrooxidans*	9.9 ± 0.7 b	7.8 ± 0.7 bc	8.3 ± 0.0 bc
S- and Fe-oxidizing microbes	1	4.7 ± 0.2 a	5.4 ± 0.1 b	6.1 ± 0.2 c	6.2 ± 0.3 c	*A. thiooxidans*	4.2 ± 0.2 a	9.0 ± 0.3 d	8.2 ± 0.1 e
S- and Fe-oxidizing microbes	2	4.7 ± 0.3 a	7 ± 0.7 b	6.0 ± 0.7 ab	5 ± 0.5 ab	*A. thiooxidans*	9 ± 1.1 b	8 ± 0.3 b	8 ± 0.3 b
S- and Fe-oxidizing microbes	3	1.0 ± 0.0 a	7.6 ± 0.5 b	10.6 ± 0.5 c	7.5 ± 0.7 b	*A. thiooxidans*	8.0 ± 0.0 b	10.1 ± 1.2 c	9.0 ± 0.0 bc
Fecal coliforms	1	6 ± 0.1	LDL	LDL	LDL	-	LDL	LDL	LDL
Fecal coliforms	2–3	LDL	LDL	LDL	LDL	-	LDL	LDL	LDL
Total coliforms	1–3	LDL	LDL	LDL	LDL	-	LDL	LDL	LDL
Enterococci	1	2.8 ± 0.2	2.1 ± 0.6	3.7 ± 0.5	3.5 ± 0.4	-	1.8 ± 0.2	3.9 ± 0.2	3.6 ± 0.1
Enterococci	2	1.0 ± 1.4	1.0 ± 0.0	3.5 ± 0.3	3.2 ± 0.1	-	2.0 ± 0.0	3.8 ± 0.3	3.3 ± 0.2
Enterococci	3	4.5 ± 1.2	LDL	LDL	LDL	-	LDL	LDL	LDL
Clostridia	1	3.5 ± 0.0	2.4 ± 0.4	3.1 ± 0.1	2.4 ± 0.1	-	2.8 ± 0.8	3.9 ± 0.2	3.6 ± 0.1
Clostridia	2	3.3 ± 0.2	1.4 ± 1.6	3.0 ± 0.1	2.3 ± 0.0	-	2.5 ± 0.7	3.8 ± 0.2	3.5 ± 0.3
Clostridia	3	LDL	LDL	LDL	LDL	-	LDL	LDL	LDL

**Table 5:** Solubilization percentage of major nutrients and pH at the end of different phases (1, 2, 3) of the experiment. The original concentrations are presented in Table 1. The symbols describing the treatments are presented in Table 3.

		Treatment						
Parameter	Phase	O	FC	TC	FTC	F	T	FT
Phosphorus	1	1	10	12	8	5	10	12
	2	0	15	5	11	10	5	5
	3	0	21	27	25	10	92	19
Sulfur	1	18	63	50	49	96	53	52
	2	26	62	54	52	65	59	45
	3	29	100	100	100	94	100	100
Potassium	1	56	ND	67	55	ND	71	63
	2	67	9	64	39	44	76	71
	3	90	1	52	21	ND	62	69
Calcium	1	21	52	45	48	80	55	57
	2	34	101	23	71	89	14	10
	3	25	87	72	75	100	70	100
Iron	1	0	59	1	56	74	1	51
	2	1	25	0	0	12	0	1
	3	0	101	71	88	39	100	74
pH	1	6.73	2.13	2.38	2.30	2.29	2.64	2.52
	2	7.00	2.62	7.04	3.45	2.38	7.72	6.59
	3	6.64	1.37	1.47	1.30	2.47	0.9	1.69

**Table 6:** Trace element concentrations in sludge before the experiments and their reduction (%) after different treatments. The symbols are given in Table 3.

Trace Element	Concentration in Sludge mg/L	Phase	Reduction % in Treatments						
			O	FC	TC	FTC	F	T	FT
Ti	1.85	1	0	33	2	14	98	12	20
	3.33	2	1	3	1	0	2	1	1
	4.51	3	0	18	5	9	10	12	15
Cr	0.60	1	0		6	100	100	4	100
	1.08	2	2	58	1	2	33	1	2
	1.46	3	0	100	67	100	116	101	100
Mn	2.80	1	1	100	58	100	100	73	100
	5.04	2	3	100	2	93	100	0	18
	6.84	3	1	100	110	100	100	100	100
Co	4.76	1	0	100	2	100	100	86	100
	8.57	2	1	77	0	2	39	0	2
	11.61	3	0	100	81	100	100	100	100
Ni	0.11	1	10	100	49	100	100	75	100
	0.20	2	25	100	12	100	100	15	9
	0.27	3	7	100	100	100	100	100	100
Cu	0.75	1	11	100	91	100	100	86	100
	1.35	2	2	100	9	51	100	4	3
	1.83	3	5	100	100	100	100	102	100
Zn	1.47	1	3	100	100	100	100	100	100
	2.64	2	1	100	2	100	100	1	1
	3.58	3	1	100	100	100	100	100	100
Br	0.05	1	60	59	59	59	61	77	68
	0.08	2	100	56	84	59	65	100	72
	0.11	3	63	83	45	55	59	41	57
Rb	0.02	1	73	47	100	100	100	100	100
	0.03	2	100	60	100	100	83	100	101
	0.04	3	51	51	100	55	37	100	37
Sr	0.23	1	11	100	77	89	100	99	100
	0.41	2	16	47	10	6	48	10	30
	0.56	3	8	100	100	100	63	100	94

## Data Availability

Not applicable.
